# A theoretical study on excited-state dynamical properties and laser cooling of zinc monohydride including spin-orbit couplings

**DOI:** 10.3389/fchem.2024.1460224

**Published:** 2024-08-20

**Authors:** Donghui Li, Faiza Fayyaz, Wensheng Bian

**Affiliations:** ^1^ Beijing National Laboratory for Molecular Sciences, Institute of Chemistry, Chinese Academy of Sciences, Beijing, China; ^2^ School of Chemical Sciences, University of Chinese Academy of Sciences, Beijing, China

**Keywords:** electronic state crossing, vibrational branching ratio, ultracold molecule, *ab initio*, spin-orbit coupling

## Abstract

By means of highly accurate *ab initio* and dynamical calculations, we identify a suitable laser cooling candidate that contains a transition metal element, namely zinc monohydride (ZnH). The internally contracted multireference configuration interaction method is employed to compute the five lowest-lying Λ-S states of ZnH with the spin-orbit coupling effects included, and very good agreement is obtained between our calculated and experimental spectroscopic data. Our findings show that the position of crossing point of the A^2^Π and B^2^Σ^+^ states of ZnH is above the *v*′ = 2 vibrational level of the A^2^Π state indicating that the crossings with higher electronic states will have no effect on laser cooling. Hence, we construct a feasible laser-cooling scheme for ZnH using five lasers based on the A^2^Π_1/2_ → X^2^Σ^+^_1/2_ transition, which features a large vibrational branching ratio *R*_00_ (0.8458), a large number of scattered photons (9.8 × 10^3^) and an extremely short radiative lifetime (64 ns). The present work demonstrates the importance of electronic state crossings and spin-orbit couplings in the study of molecular laser cooling.

## 1 Introduction

In recent years, it has attracted great research interests to establish more promising laser cooling candidates owing to their importance for a lot of potential applications such as precision measurements, quantum information storage and quantum computers (Hudson et al., 2011; Yan et al., 2013; Baron et al., 2014). Around a decade ago, the SrF molecules were successfully cooled to the micro-kelvin level using the direct laser cooling method (Shuman et al., 2010), and after that much research in molecular laser cooling has been initiated. However, so far only a few kinds of diatomic molecules have been experimentally cooled to the ultracold regime. Therefore, it is of considerable interests in searching for more promising candidates for laser cooling, and theoretical calculations could play an important role (Wells and Lane, 2011; Fu et al., 2017; Cao et al., 2019; Moussa et al., 2021; Xia et al., 2021). There are three criteria for laser cooling candidates that are generally recognized (Di Rosa, 2004): a highly diagonal Franck-Condon factor (FCF) matrix, a very short radiative lifetime, and no interference from intermediate states. In addition, Bian (Li et al., 2020) recently proposed the fourth criterion for molecular laser cooling, that is, no electronic-state crossing, or the crossing point between the two electronic states is high enough in energy relative to the ground vibrational level. Therefore, when searching for molecular candidates, all electronic states that are close to those selected for the cooling scheme should be computed and checked in advance.

Research on the spectroscopic investigation of ZnH has been ongoing since 1923, when Hulthen observed and analysed the A^2^Π → X^2^Σ^+^ transition of ZnH for the first time (Hulthen, 1923). Forty years later, there has been extensive progress in the determination of electronic states of ZnH such as C^2^Σ^+^ → X^2^Σ^+^ transition was observed and analysed by M.A. Khan (Khan, 1962) in 1962. Balfour and co-workers (Balfour et al., 1986) investigated B^2^Σ^+^ → X^2^Σ^+^ transition using high resolution photographic spectroscopy and reported the approximate FCFs for this transition in 1986. Moreover, ZnH remained a subject of FTIR and rotational spectroscopy. Hence, Shayesteh et al. (Shayesteh et al., 2006) measured the high resolution infrared emission spectra of ZnH in 2006 and Bucchino et al. (Bucchino and Ziurys, 2013) recorded pure rotational spectra, including the N = 0 → 1 and N = 1 → 2 (here N represents the rotational energy levels) transitions, for the ground state of ZnH in 2013. Bucchino and co-workers also determined the fine structure and hyperfine constants, including the Fermi contact, dipolar, and electric quadrupole parameters of Zn nuclei. Subsequently, Abbasi et al. (Abbasi and Shayesteh, 2017) detected the high-resolution emission spectra of the A^2^Π → X^2^Σ^+^ and B^2^Σ^+^ → X^2^Σ^+^ transitions for ZnH using a Fourier transform spectrometer in 2017. They obtained the Dunham coefficients, empirical band constants, and determined the purely electronic matrix elements.

Apart from experiments, ZnH has been of great interest to theoretical and computational scientists. Such as, in 1967, the spin-orbit coupling (SOC) constant of the A^2^Π state was computed for ZnH by means of the self-consistent field molecular orbital (SCF MO) theory (Ishiguro and Kobori, 1967). In 1994, Jamorski applied an averaged relativistic effective Hamiltonian method to get the spectroscopic constants of four Λ-S states and corresponding Ω states of ZnH (Jamorski et al., 1994). On the other hand, in 2009, Hayashi and co-workers (Hayashi et al., 2009) did *ab initio* study for the low-lying electronic states of ZnH and ZnH^+^. They used highly accurate multireference configuration interaction (MRCI) method with the Davidson correction (+Q) and calculated the spectroscopic constants for the bound states. Likewise, in 2017 Zhao et al. (2017) applied internally contracted MRCI+Q (icMRCI + Q) method and obtained the PECs of the seven lowest-lying Λ-S states and corresponding Ω states of ZnH. Moreover, they estimated radiative lifetimes, calculated FCFs for many transitions as well as reported the spectroscopic constants of five bound Λ-S and corresponding Ω states.

So far, as far as we know, there have not been any theoretical investigations reported on laser cooling of ZnH. In this work, by means of highly accurate *ab initio* and dynamical calculations including the SOC effects, we identify a suitable molecular candidate for laser cooling, which can fulfil the known criteria of molecular laser cooling including the fourth one proposed in our recent work. The paper is organized as follows. Section 2 briefly describes the theoretical methods and computational details. In Section 3, we discuss our calculated results, underscoring the importance of electronic state crossings and SOCs on laser cooling, and construct a feasible laser cooling scheme for ZnH. The conclusions are presented in Section 4.

## 2 Methods and computational details

In this paper, all the *ab initio* calculations of ZnH are carried out in the C_2*v*_ point group with the MOLPRO 2015.1 program package (Werner et al., 2015). The potential energies of the five Λ-S states of ZnH are computed with the complete active space self-consistent field (CASSCF) (Werner and Knowles, 1985) method. The seven-state averaged CASSCF calculations are performed for the orbital optimization, using the HF orbitals as the starting guess. Then, these CASSCF energies are taken as the reference to compute the energies of each electronic state by the icMRCI + Q method. (Langhoff and Davidson, 1974). The electrons in the 3s and 3p orbitals of the Zn atom are not included in the active space, so the core correlation on Zn is not considered in this work. It is well-known that the selection of a reasonable active space plays a crucial role in the CASSCF and MRCI + Q calculations (Liu et al., 2009; Yu and Bian, 2011; Yu and Bian, 2012). The active space of ZnH is denoted as CAS (3e, 6o) including the Zn 4s4p5s and H 1s orbitals. The outer virtual orbitals are added to give a better description on the dissociation behavior, particularly for excited electronic states (Shen et al., 2017). As for basis sets, we use the aug-cc-pwCV5Z-DK basis sets for Zn and H (Dunning and Peterson, 2000; van Mourik et al., 2000). Moreover, the SOC effects are considered by means of the state interaction approach with the Breit-Pauli Hamiltonian (*H*_BP_) in the SOC calculations (Berning et al., 2000), indicating that the SO eigenstates are acquired by diagonalizing *Ĥ*_*el*_ + *Ĥ*_*SO*_ in the basis of eigenfunctions of *Ĥ*_*el*_. Additionally, the *Ĥ*_*SO*_ and *Ĥ*_*el*_ are derived from the icMRCI + Q computations.

The Einstein spontaneous emission coefficient (*A*_*ν′ν*_) from the higher-energy state (*v*′, *J*′) to the lower-energy state (*v*, *J*) can be determined with the expression (Herzberg, 1950):

$$A_{\nu^{\prime }\nu }=3.1361891\times 10^{-7}\frac{S\left(J^{\prime },J\right)}{2J^{\prime }+1}{\Delta E}_{\nu^{\prime },\nu }^{\;3}{\left|\langle \Psi_{\nu^{\prime },J^{\prime }}\left|M\left(r\right)\right|\Psi_{\nu ,J}\rangle \right|}^{2}$$ (1)

Whereas *A*_*ν′ν*_ is expressed in units of s^-1^, $S\left(J^{\prime },J\right)$ is represented in the Hönl-London rotational intensity factor, ${\Delta E}_{\nu^{\prime },\nu }$ is the energy difference in cm^−1^ unit, $\Psi_{\nu ,J}$ and $\Psi_{\nu^{\prime },J^{\prime }}$ are the unit normalized radial wave functions, and *M* (*r*) is the transition dipole function in Debye unit, *v* and *J* are vibrational and rotational levels of the lower-energy state, respectively, and *v*′ and *J*′ are vibrational and rotational levels of the higher-energy state, respectively.

The *R*_*ν′ν*_ can be determined by the following expression:

$${\;R}_{\nu^{\prime }\nu }=A_{\nu^{\prime }\nu \;}/\sum_{v}A_{\nu^{\prime }\nu }$$ (2)

For a given vibrational level *ν*′ of an excited state, the radiative lifetime ( $\tau_{\nu^{\prime }}$ ) can be evaluated with the following expression:

$${\;T}_{Doppler}=h/\left(4k_{B}\pi \tau \right)$$ (3)

The Doppler temperature ( $T_{Doppler}$ ) which is the minimum temperature with the Doppler method for the translational cooling, can be determined with the following expression: (Fu et al., 2017):

$$T_{recoil}=h^{2}/\left(mk_{B}\lambda^{2}\right)$$ (4)

where *h* and *k*_*B*_ are Planck′s and Boltzmann′s constants, respectively, and *τ* is the radiative lifetime of the excited state.

The recoil temperature ( $T_{Doppler}$ ) can be determined with the following expression: (Li et al., 2019):

$$\tau_{\nu^{\prime }}=1/\sum_{v}A_{\nu^{\prime }\nu }$$ (5)

where *m* is the relative molecular mass and *λ* is the laser wavelength *λ*_00_.

For the bound Λ-S and Ω states of ZnH, We use the LEVEL 8.0 program (Le Roy, 2017) to determine the *A*_*ν′ν*_, FCFs, and the spectroscopic constants including the equilibrium bond length (*R*_e_), adiabatic relative electronic energy referred to the ground state (*T*_e_), the harmonic and anharmonic vibrational frequencies (*ω*_e_ and *ω*_e_ *χ*_e_), dissociation energy (*D*_e_) and the rotational constant (*B*_e_).

## 3 Results and discussion

### 3.1 PECs and molecular spectroscopic constants

**FIGURE 1:**
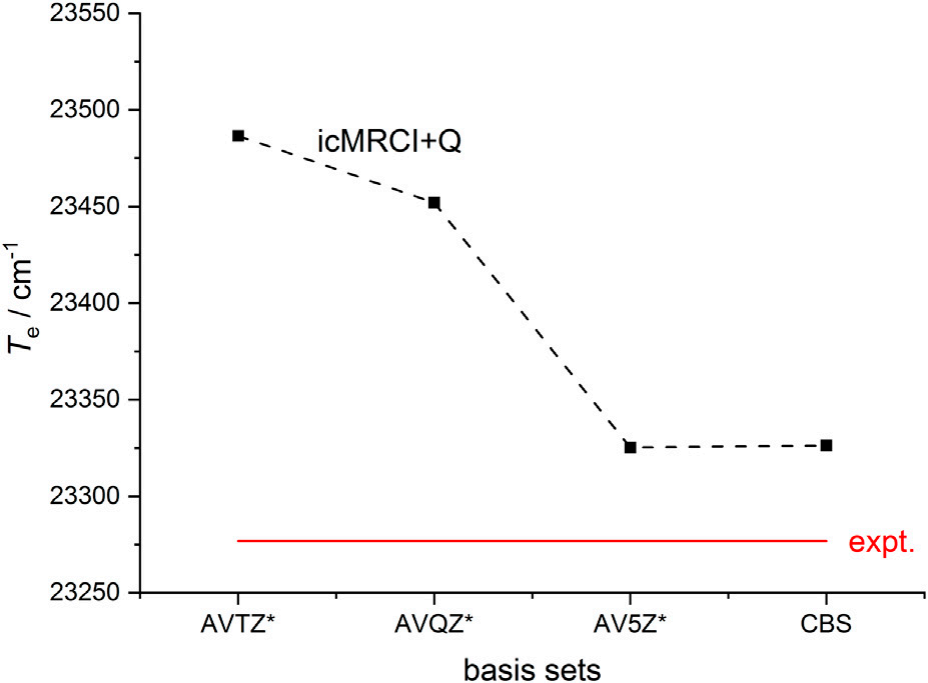
The convergence pattern of the adiabatic relative electronic energy relative to the ground state (*T*_e_) of the A^2^Π state for ZnH toward the estimated complete basis set (CBS) limit using the icMRCI(3e, 6o) + Q method with the AVnZ* (n = T, Q, 5) basis sets. AVnZ* represents aug-cc-pwCVTZ-DK, aug-cc-pwCVQZ-DK and aug-cc-pwCV5Z-DK respectively. The corresponding experimental value (Huber and Herzberg, 1979) is also shown.

**FIGURE 2:**
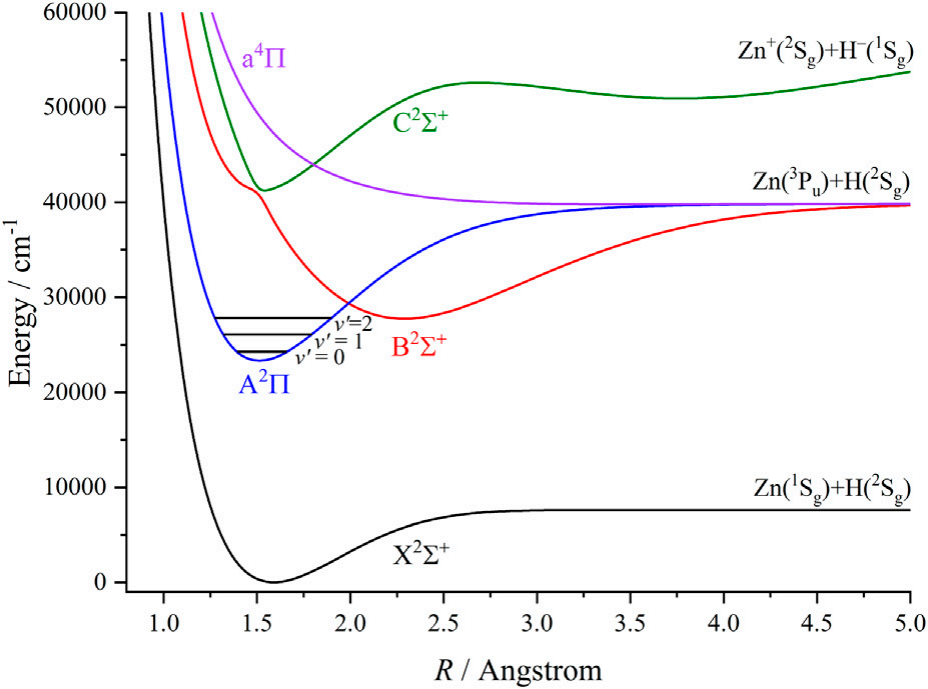
Potential energy curves of ZnH as a function of the interatomic distance (*R*) for the five lowest-lying Λ-S states at the icMRCI + Q level.

The icMRCI + Q calculations are carried out to check the convergence of the computed results with distinct basis sets (the aug-cc-pwCVTZ-DK, aug-cc-pwCVQZ-DK and aug-cc-pwCV5Z-DK basis sets are denoted as AVTZ, AVQZ and AV5Z, respectively), and the calculated *T*_e_ results of the A^2^Π state are shown in Figure 1. Here the complete basis set (CBS) energy is evaluated by a three-point extrapolation scheme. (Peterson et al., 1994; Koput and Peterson, 2002; Peterson and Dunning, 2002). The plotted curve in Figure 1 indicates that the result obtained by the AV5Z (aug-cc-pwCV5Z-DK) basis sets is very close to the CBS result, and in very good agreement with the experimental value. (Huber and Herzberg, 1979). We conclude that the aug-cc-pwCV5Z-DK basis set is large enough and thus is used for further calculations in this work. Figure 2 depicts the PECs of the five lowest-lying Λ-S electronic states of ZnH, derived from the icMRCI + Q calculations. As can be seen, the ground state X^2^Σ^+^ converges towards the lowest neutral atomic Zn (^1^S_g_) + H (^2^S_g_) limit, while the A^2^Π, B^2^Σ^+^ and a^4^Π states correlate with the Zn (^3^P_u_) + H (^2^S_g_) limit. Additionally, the C^2^Σ^+^ state corresponds to the Zn^+^(^2^S_g_) + H^−^(^1^S_g_) limit. In this work the A^2^Π state is used to establish laser cooling cycles for ZnH, and thus we did not consider much higher excited states (e.g., the 1^4^Σ^+^ state), which will not influence our discussion. As the values of spectroscopic constants for the low-lying bound states of ZnH are already determined experimentally and reported in literature, we compared our computed results with these data that could verify the accuracy and reliability of our calculations. Table 1 provides a comparison of our computed and already available spectroscopic constants values of the three bound Λ-S states of ZnH. For the first excited state A^2^Π, the experimentally determined value (Huber and Herzberg, 1979) is 23276.9 cm^-1^ whereas our computed value is 23325.25 cm^−1^, using larger basis set. In contrast, the previously computed value (Zhao et al., 2017) at smaller basis sets (aug-cc-pwCVTZ-DK) is 23469 cm^-1^, deviated from experimental value by approximately 190 cm^-1^. Furthermore, our computed spectroscopic constants, i.e., *R*_e_, *ω*_e_, *ω*_e_ *χ*_e_ and *B*_e_ values of the A^2^Π state and *R*_e_, *ω*_e_, *ω*_e_ *χ*_e,_ *D*_*e*_ and *B*_e_ values of the ground state X^2^Σ^+^ are very close to the experimental values (Huber and Herzberg, 1979). Moreover, our computed *R*_e_, *ω*_e_, *ω*_e_ *χ*_e_ and *B*_e_ values of the B^2^Σ^+^ state are highly consistent to the measurements (Huber and Herzberg, 1979). Over all, it can be seen that our results align very well with the experimental data (Huber and Herzberg, 1979), though the deviation in the *T*_e_ value for the B^2^Σ^+^ between our calculated data and the experimental result is slightly larger than that from the previous calculations (Zhao et al., 2017). It is worth noting that accurate determination of the *T*_e_ value for the A^2^Π state plays a pivotal role in obtaining the laser wavelengths for laser-driven cycling. In this regard, our calculated *T*_e_ values match well with corresponding experimental results, raising the confidence to further investigate laser cooling of ZnH.

**TABLE 1:** Spectroscopic constants of the three ʌ-S states for ZnH.

State	Method	*T*_e_ (cm^−1^)	*R*_e_ (Å)	*ω*_e_ (cm^−1^)	*ω*_e_ *χ*_e_ (cm^−1^)	*D*_e_ (eV)	*B*_e_ (cm^−1^)
X^2^Σ^+^	This work	0	1.592	1603.27	53.57	0.9420	6.7054
	Expt.^a^	0	1.5949	1607.6	55.14	0.91	6.6794
	Calc.^b^	0	1.5926	1600	51.11		6.7026
A^2^Π	This work	23325.25	1.512	1907.25	41.14	2.0488	7.4279
	Expt.^a^	23276.9	1.5119	1910.2	40.8		7.4332
	Calc.^b^	23469	1.514	1903	40.57	2.02	7.4129
B^2^Σ^+^	This work	27740.86	2.290	1018.27	16.43	1.4308	3.2450
	Expt.^a^	27587.7	2.273	1020.7	16.5	1.40	3.288
	Calc.^b^	27584.15	2.293	1006	16.62	1.42	3.2323

^a^ Reference (Huber and Herzberg, 1979).

^b^ Reference (Zhao et al., 2017).

**FIGURE 3:**
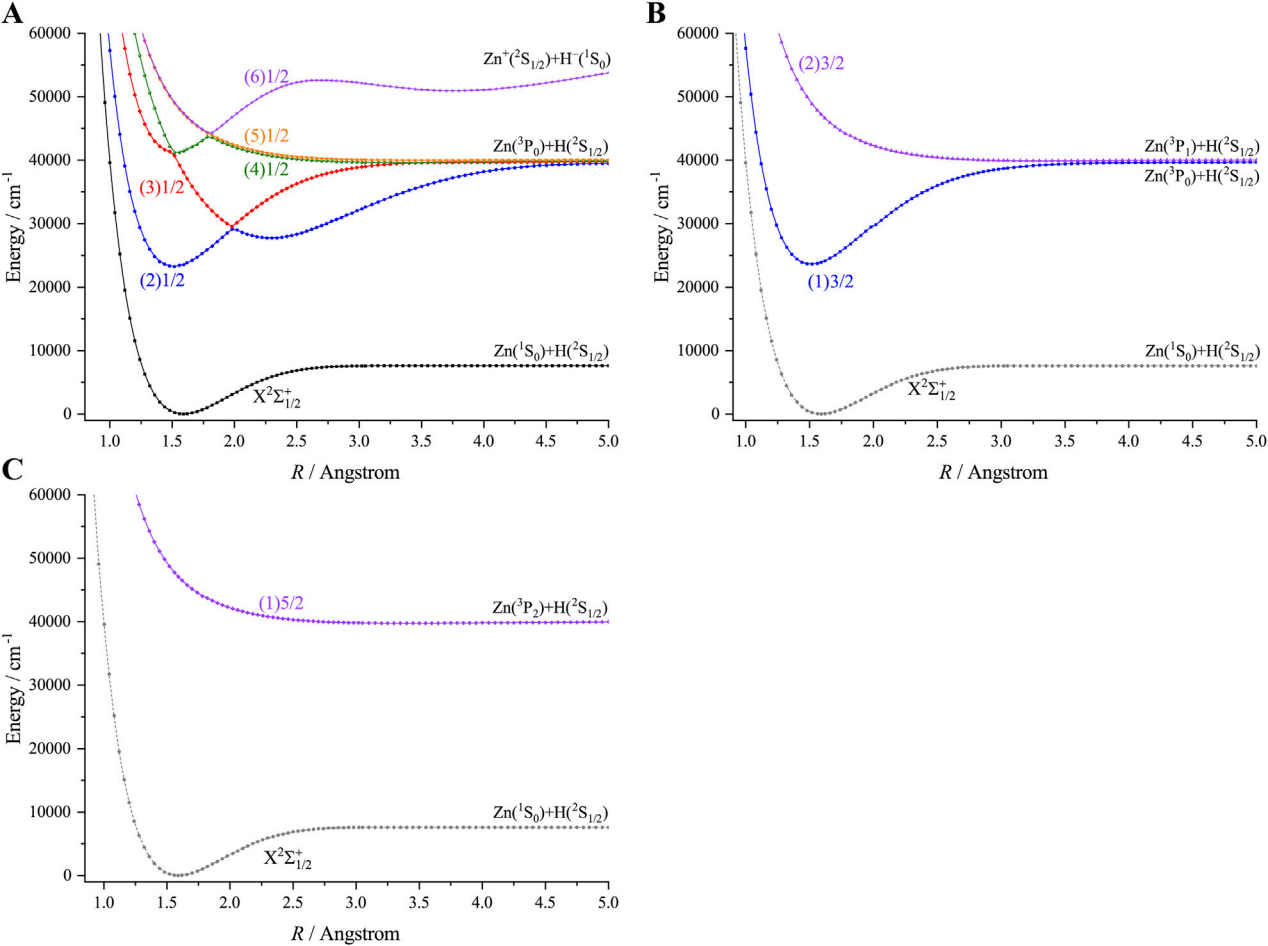
Potential energy curves of ZnH as a function of the interatomic distance (R) for **(A)** Ω = 1/2, **(B)** Ω = 3/2 and **(C)** Ω = 5/2 at the icMRCI + Q level.

Inclusion of the SOC effects has resulted in the splitting of five Λ-S states (X^2^Σ^+^, A^2^Π, B^2^Σ^+^, C^2^Σ^+^ and a^4^Π) of ZnH into 9 Ω states, of which 6 states have Ω = 1/2 (X^2^Σ^+^_1/2_, A^2^Π_1/2_, B^2^Σ^+^_1/2_, C^2^Σ^+^_1/2_, a^4^Π_1/2_ and (2) a^4^Π_1/2_), two states have Ω = 3/2 (A^2^Π_3/2_ and a^4^Π_3/2_), and one state has Ω = 5/2 (a^4^Π_5/2_). The PECs of the 9 Ω states of ZnH are depicted in Figure 3. The spectroscopic constants of the 4 Ω states of ZnH, namely, X^2^Σ^+^_1/2_, A^2^Π_1/2_, A^2^Π_3/2_ and B^2^Σ^+^_1/2_, are presented in Table 2. As can be seen, the spectroscopic constants of the 2 Ω states X^2^Σ^+^_1/2_ and B^2^Σ^+^_1/2_ are very close to those of Λ-S states of ZnH. In addition, our calculated SO splitting value of the A^2^Π state (331.84 cm^−1^) is in very good agreement with the experimental value (Huber and Herzberg, 1979) (342.66 cm^−1^) and outperforms the previous theoretical result (Zhao et al., 2017) (241.96 cm^−1^). It is evident that SO splitting value of the A^2^Π state is relatively large and indicates that the SOC effects should be considered while studying the excited states of ZnH. Hence, SOC effects are important for laser cooling of ZnH.

**TABLE 2:** Spectroscopic constants of the 4 Ω states for ZnH.

State	Method	*T*_e_ (cm^−1^)	*R*_e_ (Å)	*ω*_e_ (cm^−1^)	*ω*_e_ *χ*_e_ (cm^−1^)	*D*_e_ (eV)	*B*_e_ (cm^−1^)
X^2^Σ^+^_1/2_	This work	0	1.592	1602.24	53.68	0.9422	6.7065
	Expt.^a^	0	1.5949	1607.6	55.14	0.91	6.6794
	Calc.^b^	0	1.5926	1600	51.05		6.7030
A^2^Π_1/2_	This work	23280.05	1.512	1909.69	41.27	2.0425	7.4317
	Expt.^a^	23276.9	1.5119	1910.2	40.8		7.4332
	Calc.^b^	23390.07	1.5135	1911	44.1	1.94	7.4179
A^2^Π_3/2_	This work	23611.89	1.512	1909.55	41.02	1.9917	7.4271
	Calc.^b^	23632.03	1.5141	1902	40.5	1.97	7.4096
B^2^Σ^+^_1/2_	This work	27739.10	2.290	1018.35	16.47	1.4219	3.2447
	Expt.^a^	27587.7	2.273	1020.7	16.5	1.40	3.288
	Calc.^b^	27584.15					

^a^ Reference (Huber and Herzberg, 1979).

^b^ Reference (Zhao et al., 2017).

### 3.2 The effects of the electronic state crossings and spin-orbit couplings

From Figure 2, we can see that the A^2^Π and B^2^Σ^+^ states of ZnH have a crossing point, which can lead to nonradiative transition (Wu et al., 2019), and result in predissociation. It is evident that, for polyatomic molecules, this type of electronic state crossings will become potential energy surface intersections (Liu et al., 2003; Zhao et al., 2006). We can see that the location of crossing point between the A^2^Π and B^2^Σ^+^ states of ZnH is above the *ν*′ = 2 vibrational level of the A^2^Π state (1540 cm^-1^) indicating that the crossings with higher electronic states will not affect laser cooling. With the inclusion of the SOC effects, the A^2^Π state will split into two states (A^2^Π_1/2_ and A^2^Π_3/2_), and the potential energy of the A^2^Π_1/2_ state is a little lower than that of A^2^Π_3/2_. Thus we will use the A^2^Π_1/2_ → X^2^Σ^+^_1/2_ transition to construct a laser cooling scheme for ZnH. Consequently, hereafter, a feasible laser cooling scheme for ZnH is constructed on the basis of the A^2^Π_1/2_ → X^2^Σ^+^_1/2_ transition, which meets the known criteria including the fourth one proposed in our recent work (Li et al., 2020).

### 3.3 Laser cooling scheme proposed for ZnH using specific spin-orbit states

Given the significance of the SOC effects as demonstrated above, we construct a laser cooling scheme for ZnH based on the A^2^Π_1/2_ → X^2^Σ^+^_1/2_ transition, which is free from interference by any intermediate state. In this scheme, ZnH molecules are initially excited from the X^2^Σ^+^_1/2_ (*ν* = 0) state to the A^2^Π_1/2_ (*ν*′ = 0) state, then they decay back to the X^2^Σ^+^_1/2_ state. This will generate ultracold ZnH molecules when the cooling cycles are consistently repeated.

**FIGURE 4:**
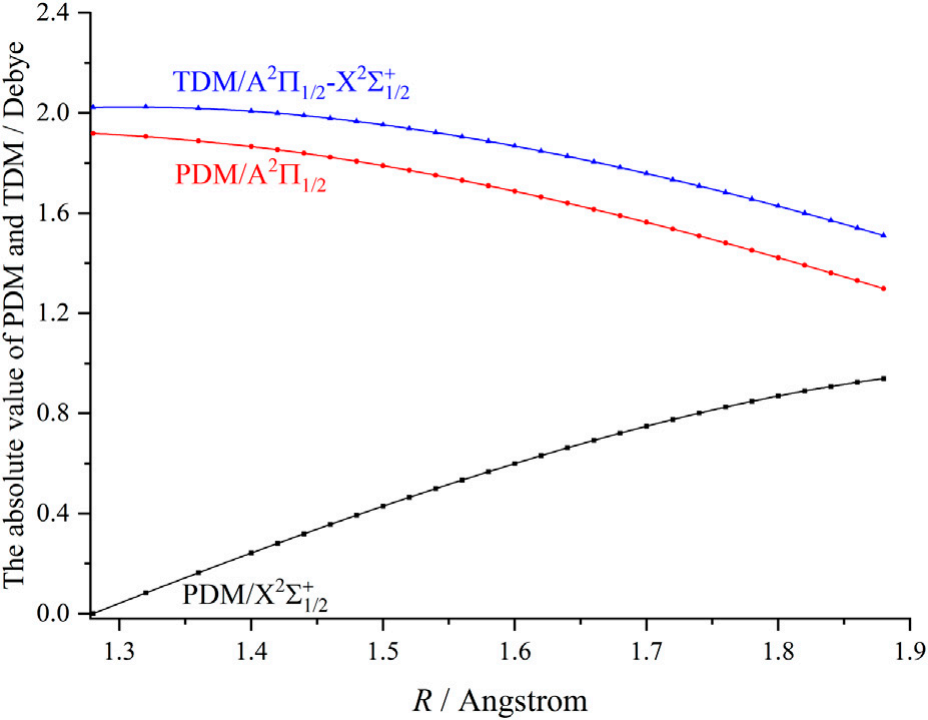
The computed permanent dipole moments (PDMs) and transition dipole moments (TDMs) for the X^2^Σ^+^_1/2_ and A^2^Π_1/2_ states of ZnH at the icMRCI + Q level.

Figure 4 presents the permanent dipole moments (PDMs) and transition dipole moments (TDMs) for the A^2^Π_1/2_ → X^2^Σ^+^_1/2_ transition of ZnH at the icMRCI + Q level. As can be seen, the TDMs of ZnH gradually decrease with the increasing bond length, reaching 1.88 debye at *R*_e_. In addition, our computed FCF (*f*_*ν*′*ν*_) value of the A^2^Π_1/2_ (*ν*′ = 0) → X^2^Σ^+^_1/2_ (*ν* = 0) transition (or the *f*_00_ value) for ZnH is 0.8367. This relatively large *f*_00_ value indicates that the spontaneous decays to *ν* = 1–4 vibrational levels of the X^2^Σ^+^_1/2_ state are highly suppressed. Based on the A^2^Π_1/2_ → X^2^Σ^+^_1/2_ transition, we use the *ν*′ = 0, 1 levels of the A^2^Π_1/2_ state of ZnH with five lasers to establish laser cooling scheme. Furthermore, we have computed and tabulated the Einstein A coefficients (*A*_*ν*′*ν*_, Equation 1) and vibrational branching ratios (*R*_*ν*′*ν*_, Equation 2) values of the A^2^Π_1/2_ → X^2^Σ^+^_1/2_ transition (see Table 3), since the relative strengths of photon loss channels are directly linked to the *R*_*ν*′*ν*_ rather than the *f*_*ν*′*ν*_ in laser cooling process. A very large *A*_00_ value of 1.32×10^7^ s^-1^ and very minimal scattering probabilities into off-diagonal bands provide favourable conditions for rapid and efficient laser cooling cycles.

**TABLE 3:** Calculated Einstein A coefficients *A*_*ν*′*ν*_ and vibrational branching ratio *R*_*ν*′*ν*_ of the A^2^Π_1/2_ (*ν′*) → X^2^Σ^+^_1/2_ (*ν*) transition for ZnH.

	*ν'* = 0		*ν'* = 1		*ν'* = 2	
	*A*_*ν*′*ν*_	*R*_*ν*′*ν*_	*A*_*ν*′*ν*_	*R*_*ν*′*ν*_	*A*_*ν*′*ν*_	*R*_*ν*′*ν*_
*ν* = 0	1.32 × 10^7^	0.8458	2.35×10^6^	1.55 × 10^−2^	8.23 × 10^4^	5.80 × 10^−3^
*ν* = 1	2.11 × 10^6^	1.35 × 10^−1^	8.19×10^6^	0.5419	4.16 × 10^6^	2.93 × 10^−1^
*ν* = 2	2.59 × 10^5^	1.66 × 10^−2^	3.58×10^6^	2.37 × 10^−1^	3.89 × 10^6^	2.74 × 10^−1^
*ν* = 3	3.28 × 10^4^	2.10 × 10^−3^	7.95×10^5^	5.26 × 10^−2^	4.03 × 10^6^	2.84 × 10^−1^

In addition, the evaluated Doppler and recoil temperatures (*T*_*Doppler*_ and *T_recoil_* can be evaluated with the Equations 3 and 4, respectively) for the A^2^Π_1/2_ (*ν*′ = 0) → X^2^Σ^+^_1/2_ (*ν* = 0) transition of ZnH are 59.73 *µK* and 1.59 *µK*, respectively. The computed radiative lifetime ( $\tau_{\nu^{\prime }}$ , Equation 5) for the main cooling transition of ZnH is 64 ns, which is extremely short and ensures a rapid laser cooling process.

**FIGURE 5:**
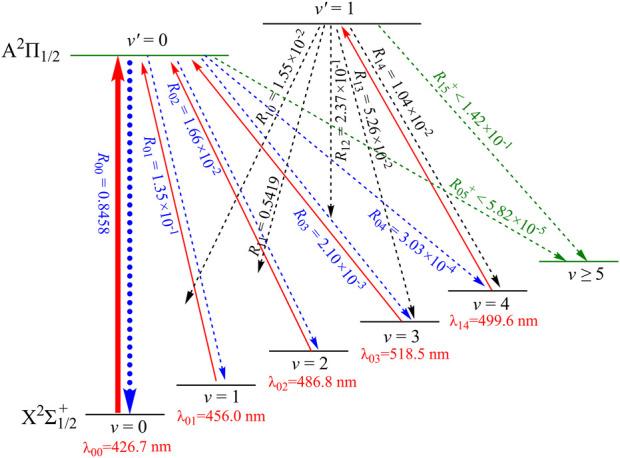
Constructed five-laser cooling scheme for ZnH using the A^2^Π_1/2_ (*ν*′) → X^2^Σ^+^_1/2_ (*ν*) transitions. Solid arrows indicate laser-driven transitions at certain wavelengths *λ*_*ν*′*ν*_. Dashed arrows indicate spontaneous decays from the A^2^Π_1/2_ (*ν*′ = 0, 1) states with the calculated vibrational branching ratios.

Our constructed five-laser cooling scheme for the production of ultracold ZnH is depicted in Figure 5. As illustrated, the laser wavelength for the main cycling will drive the X^2^Σ^+^_1/2_ (*ν* = 0, *J* = 1) → A^2^Π_1/2_ (*ν*′ = 0, *J′* = 0) transition of ZnH at the wavelength *λ*_00_ of 426.7 nm (here *J* means the rotational quantum number). According to the angular momentum and parity selection rules, the A^2^Π_1/2_ (*ν*′ = 0, *J′* = 0) state can only decay back to the initial X^2^Σ^+^_1/2_ (*ν* = 0, *J* = 1) state, thereby eliminating rotational branching. Additionally, to minimize vibrational branching losses, another 4 repump lasers are employed to recover the molecules that have decayed to the X^2^Σ^+^_1/2_ (*ν* = 1, 2, 3, 4) states of ZnH. Consequently, quasi-closed cooling cycles could be achieved using the constructed cooling scheme as presented in Figure 5. Furthermore, the calculated *R*_00_ value of ZnH is 0.8458, indicating that the A^2^Π_1/2_ (*ν*′ = 0) → X^2^Σ^+^_1/2_ (*ν* = 0) transition of ZnH has the largest possibility, whereas the vibrational branching loss should be solved in laser cooling cycle process. Thus the off-diagonal *R*_*ν'ν*_ of ZnH are also computed. In addition, we use $R_{05^{+}}+R_{04}\times R_{15^{+}}$ (here 5 ^+^ means *ν* ≥ 5 levels) to access the possibilities of the undesirable decay paths for ZnH. The negligible value of 1.01 × 10^−4^ indicates that following the present cooling scheme, ZnH on average, can scatter at least 9.8×10^3^ photons, which are sufficient for cooling ZnH to ultracold temperatures, in principle (Shuman et al., 2010). In our constructed five-laser cooling scheme for ZnH, the X^2^Σ^+^_1/2_ (*ν* = 0) → A^2^Π_1/2_ (*ν*′ = 0) transition is the main pump. While the X^2^Σ^+^_1/2_ (*ν* = 1, 2, 3) → A^2^Π_1/2_ (*ν*′ = 0) and X^2^Σ^+^_1/2_ (*ν* = 4) → A^2^Π_1/2_ (*ν*′ = 1) transitions are the secondary vibrational repumps. In the constructed laser-driven cycling for ZnH, all the calculated laser wavelengths fall in the visible range, making them readily accessible.

## 4 Conclusions

In this work, the five lowest-lying Λ-S states of ZnH are investigated by means of highly accurate *ab initio* and dynamical calculations including the SOC effects. Our computational results agree very well with experimental spectroscopic data. In addition, we study direct laser cooling of ZnH, and reveal the effects of electronic state crossings and SOC. Our calculations indicate that ZnH is a suitable candidate for laser cooling, which can fulfil the known criteria including the fourth one proposed in our recent work. The position of crossing point between the A^2^Π and B^2^Σ^+^ states of ZnH is higher than the *ν*′ = 2 vibrational level of the A^2^Π state indicating that the crossings with higher electronic states will not affect the laser cooling. Hence, we construct a practical laser-cooling scheme for ZnH based on the A^2^Π_1/2_ → X^2^Σ^+^_1/2_ transition. Our calculated excitation energy to the A^2^Π state of ZnH is 23325.25 cm^-1^, which closely matches with the experimental value of 23276.9 cm^-1 38^. This enables us to determine laser wavelengths in laser-cooling cycles accurately. The Doppler and recoil temperatures for the main transition of ZnH are 59.73 *µK* and 1.59 *µK*, respectively. The vibrational branching ratios (*R*_*ν*′*ν*_) for the A^2^Π_1/2_ (*ν*′ = 0) → X^2^Σ^+^_1/2_ transition of ZnH are shown to be diagonally distributed with *R*_00_ being 0.8458. The radiative lifetime for the A^2^Π_1/2_ (*ν*′ = 0) → X^2^Σ^+^_1/2_ (*ν* = 0) transition of ZnH is extremely short (64 ns). The present constructed scheme is able to scatter 9.8 × 10^3^ photons for ZnH, which are sufficient for cooling ZnH to ultracold temperatures. It is expected that this theoretical study will encourage for the experimental laser cooling of ZnH to ultracold temperatures.

## Data Availability

The original contributions presented in the study are included in the article/supplementary material, further inquiries can be directed to the corresponding author.
